# Active control of broadband sound through the open aperture of a full-sized domestic window

**DOI:** 10.1038/s41598-020-66563-z

**Published:** 2020-07-09

**Authors:** Bhan Lam, Dongyuan Shi, Woon-Seng Gan, Stephen J. Elliott, Masaharu Nishimura

**Affiliations:** 1 School of Electrical and Electronic Engineering, Nanyang Technological University, Singapore, 639798 Singapore; 2 Institute of Sound and Vibration Research, University of Southampton, Southampton, SO17 1BJ United Kingdom; 3 N.Lab., 1568-10, Fujie, Akashi, 673-0044 Japan

**Keywords:** Electrical and electronic engineering, Mechanical engineering

## Abstract

Shutting the window is usually the last resort in mitigating environmental noise, at the expense of natural ventilation. We describe an active sound control system fitted onto the opening of the domestic window that attenuates the incident sound, achieving a global reduction in the room interior while maintaining natural ventilation. The incident sound is actively attenuated by an array of control modules (a small loudspeaker) distributed optimally across the aperture. A single reference microphone provides advance information for the controller to compute the anti-noise signal input to the loudspeakers in real-time. A numerical analysis revealed that the maximum active attenuation potential outperforms the perfect acoustic insulation provided by a fully shut single-glazed window in ideal conditions. To determine the real-world performance of such an active control system, an experimental system is realized in the aperture of a full-sized window installed on a mockup room. Up to 10-dB reduction in energy-averaged sound pressure level was achieved by the active control system in the presence of a recorded real-world broadband noise. However, attenuation in the low-frequency range and its maximum power output is limited by the size of the loudspeakers.

## Introduction

Finding a sustainable and practical solution for controlling noise entering into naturally ventilated buildings is a difficult problem, especially for densely-populated, tropical, high-rise cities^1,2^. Due to the impracticality of erecting noise barriers for high-rise buildings, façade elements play an especially critical role in the mitigation of urban noise. Ironically, the demand for naturally ventilated buildings is exacerbating the noise problem by providing more points of entry. As outlined by De Salis and recently updated by Tang, strategies for noise control in naturally-ventilated buildings are predominantly passive, whereby physical structures are employed to disrupt the propagation path of the noise prior to entry into the room interior^1,2^. In the context of dense high-rise cities, only the plenum window strategy has shown promise^3,4^, but it has yet to overcome its inherent reduction in natural ventilation^2^. Proposed noise mitigation solutions for fully-opened apertures have thus far been largely based on active control techniques^5–10^.

An active noise control (ANC) system is an electroacoustic system, which usually comprises of a ‘reference’ sensor to provide advance information of the primary noise to be attenuated, an actuator driven by an adaptive circuit to produce the anti-noise, and an ‘error’ sensor to provide feedback to the adaptive circuit to adapt to changes in the primary noise. Although control is most effective at source, it is usually infeasible for most scenarios. Therefore, noise propagating through air is often attenuated by sound pressure reduction at the error sensor position. In a diffused field, e.g. in a car cabin, reduction of sound pressure at a few error microphones will result in a ‘local’ quiet zone around each microphone up to a tenth of a wavelength of the upper limit of control, e.g. 3.4 cm for control up to 1 kHz^11^. Hence, for a large interior space, numerous error microphones must be distributed within the interior space to achieve ‘global’ control, e.g. in propeller aircraft^12,13^ and automobiles^14^. Providentially, noise propagating through an open aperture can be treated as the source to be controlled, and both the control sources and error microphones could be arranged optimally to achieve global control by minimising the total sound power output of the aperture, as shown in a previous numerical study^15^.

Ideally, the anti-noise generating loudspeakers should be distributed around the boundary of the window to minimize visual obstruction as demonstrated by the double-layered virtual barrier system^6^. The virtual barrier system was designed to attenuate noise through a baffled rectangular opening from the interior of a short duct, mirroring industrial ventilation ducts. The performance of the boundary-based virtual barrier system is however, physically limited by the size of the opening, which restricts its suitability for regular windows^16^. Numerical simulations have shown that the boundary-based layout is ineffective for an aperture in a thin rigid wall, i.e. window on the building façade^5^.

This paper describes a system in which a planar array of control loudspeakers – distributed across the opening of a full-sized, two-panel sliding window – is driven to attenuate broadband noise impinging into the room interior. Most of the previous implementations have been installed on small, non-standard window apertures without window panels^8,17^. Even though a similar setup has been recently described for the active control of tonal noise^5^, the demands of broadband noise control on a larger aperture demanded a higher channel-count system that had to be realised on a different computing platform with major modifications. The active attenuation potential of the modified system in comparison to full glazing acoustic insulation is described in the supplementary data. Our experiments show that up to 10-dB reduction in energy-averaged sound pressure level is attainable in the frequency range of typical urban transportation noise^18^, with a fully-opened two-panel sliding window.

## Results

**Figure 1:**
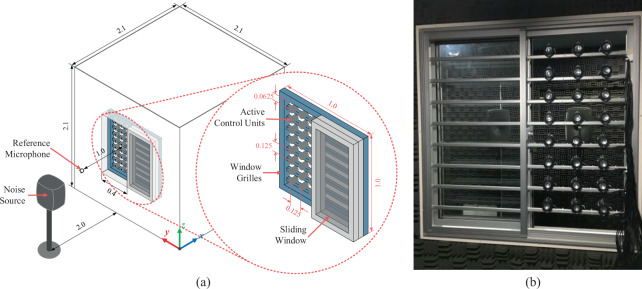
(**a**) Schematic of the mock-up room, and (**b**) view of the active control system from the inside of the mock-up chamber.

A 1 m × 1 m wide two-pane sliding window was installed on a mock-up room made from 6 panels of 30 mm thick plywood with a dimension of 2.1 m × 2.1 m × 2.1 m. A 24-channel ANC system comprised of 24 individual control units was installed on the security grille affixed to the window, a common feature in Southeast Asia, as shown in Fig. 1(a). Each control unit comprises of one loudspeaker facing the interior of the mock-up room, as detailed in Fig. 1(b). The primary source, a loudspeaker that emits the noise to be attenuated by the proposed system, is placed 2 m away from the window aperture. After accounting for the window and grille frames, the open area measures 0.45 m × 0.93 m. The control units (with 4.5 cm diameter loudspeakers) are spaced 0.125 m apart with the units at the periphery placed 0.0625 m away from the edge of the aperture. A single reference microphone, used to detect the impinging noise from the primary source, was positioned 1 m from the noise source in the middle of the 24-channel control source array, as shown in Fig. 1(a). The experimental setup is guided by 2D finite element method (FEM) simulations (see^15^ and Supplementary Material online), which investigated the passive acoustic attenuation provided by full glazing and the active attenuation of an ANC system in combination with sliding glass panels. At least 10 dB of attenuation is attainable up to 1 kHz with three active “line” sources in the half-open aperture, which resembles the fully-opened two-pane sliding window with three columns of active sources, as shown in Fig. 1(b). Under ideal conditions, the passive acoustic attenuation of full glazing appears to plateau after about 1 kHz. Moreover, from 75% glazing with three active sources, attenuation exceeds the passive insulation of fully glazing up to 1 kHz. Hence, good active control is expected below 1 kHz for the experimental system.

The global attenuation performance of the active control system was evaluated in the experiments based on the energy-averaged sound pressure level (SPL) in the room interior with an array of 7 observation microphones arranged in accordance to ISO 16283–3^19^. The energy-average SPL represents the space and time average of the indoor sound pressure level that negates the influence of nearfield radiation from the room boundaries and avoids the acoustical modes in the room^19^. As a comparison, the average SPL of a plane array of 12 microphones, denoted as planar-average SPL, positioned 0.5 m away from the window was evaluated. The planar-average corresponds to the error microphone positions where the sum-of-the-squared sound pressures were minimized by the active control system. A total of 18 microphones were utilised in both the energy-average and planar-average calculations, which is described in detail in the Methods section.

To investigate the practicality of an ANC system for domestic windows, a fixed-filter approach, where error microphones were removed during operation, was adopted. A bandlimited white gaussian noise signal (BLWN) from 100 Hz to 1 kHz was the primary noise to be controlled in an initial training stage. After steady-state control of the BLWN signal, the filter coefficients were stored and will be utilized to perform active control on other noise samples of the same bandwidth. The attenuation performance of the active control system is benchmarked to the passive attenuation provided by fully closing the two-pane sliding window. The active control performance is evaluated on a representative set of urban transportation noise types. Samples of highway noise, elevated mass rapid transit (MRT) train noise, and jet aircraft fly-by noise were recorded at the windows of a high-rise residential apartment building in Singapore.

The passive attenuation provided by the closure of the sliding window is between 12.36 dB to 13.9 dB for energy-average SPL and between 14.42 dB to 16.23 dB for planar average SPL, as shown in Table 1. The active attenuation ranges between 7.51 dB to 10.14 dB for energy-average SPL, and between 8.7 dB to 11.44 dB for planar-average SPL. Since the difference in active attenuation levels between the planar-average and energy-average SPL was 0.98 dB on average, global attenuation was achieved in the room interior.

**Table 1:** A-weighted energy-average sound pressure level of bandlimited urban transport noise recordings before and active control with windows fully opened, and without active control with windows fully closed. Values inside the parentheses indicate the attenuation level.

Noise Type (Bandwidth, Hz)	Duration, s	Energy-average SPL, dBA			Planar-average SPL, dBA		
		Before control	After control (Attenuation)	Passive (Attenuation)	Before control	After control (Attenuation)	Passive (Attenuation)
Gaussian white noise (100 to 1000)	10	74.60	65.80 (8.80)	61.80 (12.8)	79.90	70.30 (9.60)	65.21 (14.69)
Highway noise (100 to 1000)	6.64	72.93	64.26 (8.67)	60.02 (12.91)	78.20	68.95 (9.25)	63.62 (14.58)
MRT noise (100 to 1000)	12.64	77.47	67.33 (10.14)	65.11 (12.36)	82.59	71.15 (11.44)	68.17 (14.42)
Aircraft fly-by noise (100 to 1000)	19.11	70.69	63.18 (7.51)	56.79 (13.9)	75.88	67.18 (8.7)	59.65 (16.23)

**Figure 2:**
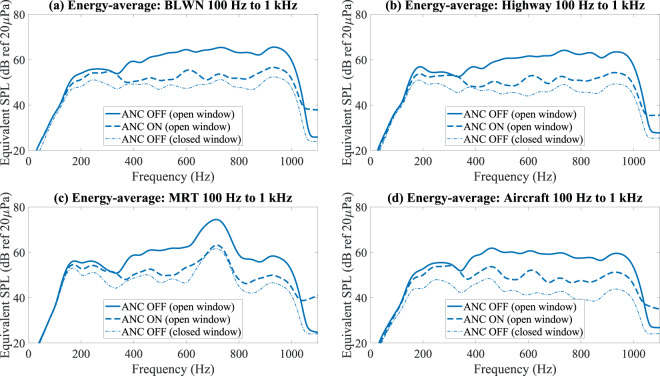
A-weighted energy-average spectrum of 100 Hz to 1 kHz band-limited (**a**) gaussian white noise, (**b**) highway noise, **(c**) MRT pass-by noise, and (**d**) aircraft fly-by noise, before active control , after active control , and with windows fully shut without active control .

**Figure 3:**
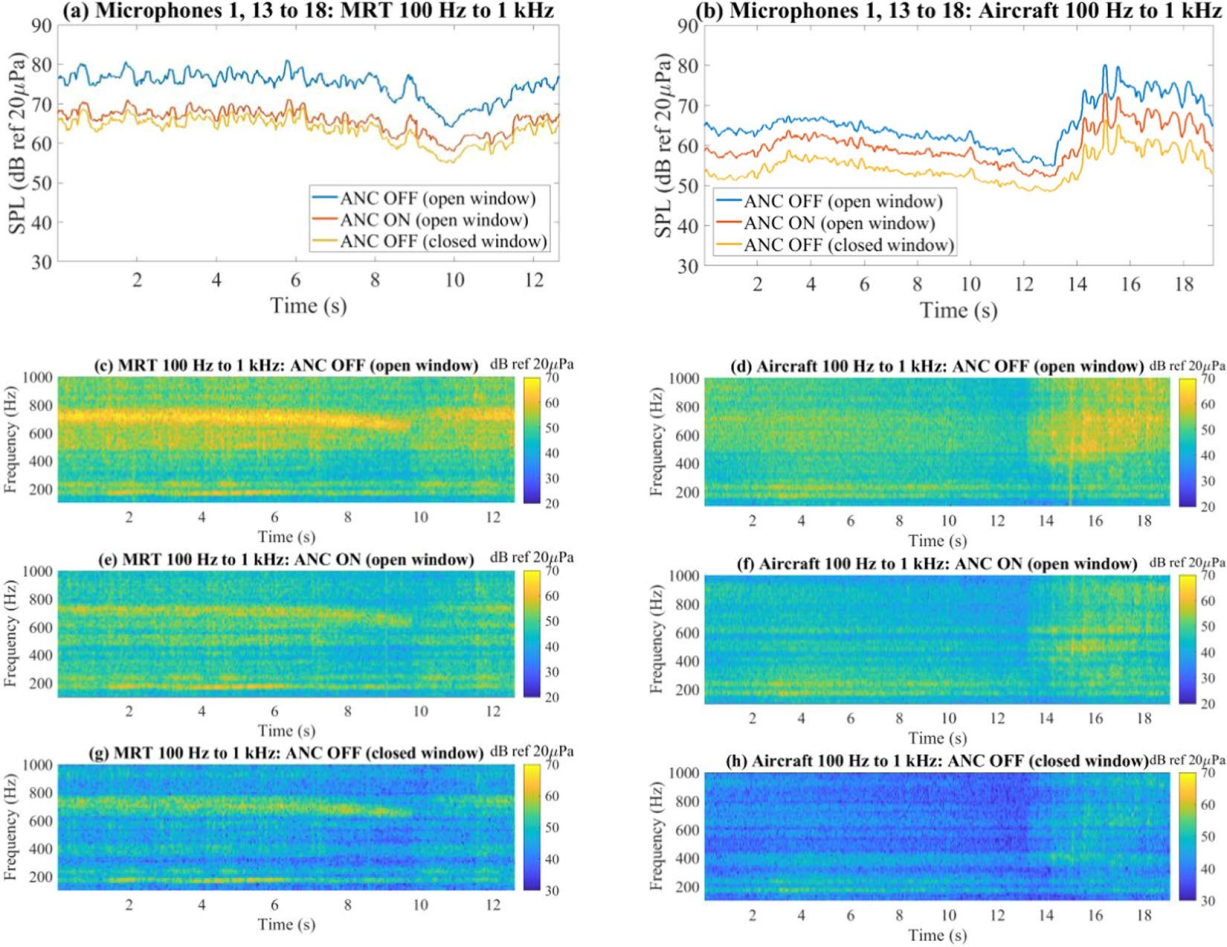
Moving-average sound pressure level as a function of time before active control , after active control , and with windows fully shut without active control . Cases shown for 100 Hz to 1 kHz bandlimited (**a**) MRT pass-by noise, and (**b**) aircraft fly-by noise. The spectrograms before control (**c,d**), after control **(e**,**f**), and windows fully closed (**g,h**) are shown for MRT and aircraft noise, respectively.

The A-weighted energy-average spectra of the noise samples before and after active control with windows fully opened, and without active control with the windows fully closed, are shown in Fig. 2. Below 300 Hz, passive attenuation is about 5 dB and almost no active control was observed for all noise samples. Active and passive control is slightly restricted from 300 to 500 Hz. Beyond 500 Hz, about 10 dB to 12 dB of passive attenuation is observed, and active attenuation of about 10 dB is achieved for all noise samples. The difference in active control performance between the time-varying MRT and aircraft fly-by noise samples were further analysed in the time domain, as shown in Fig. 3(a,b), respectively. Despite some amplitude fluctuations in the MRT and aircraft noise, the moving-average pressure measured by the 7 microphones (for energy-average SPL calculations) indicate a uniform attenuation over time. A higher attenuation was achieved for MRT noise due to a substantial reduction in the dominant noise at 700 Hz, as illustrated by the spectrograms in Fig. 3(c,e), whereas the dominant energy for aircraft noise is distributed from 400 Hz to 1 kHz, as shown in Fig. 3(d). The prominent residual aircraft noise around 500 Hz after active control exposes the restriction in control between 300 Hz to 500 Hz, as shown in Fig. 3(f). The spectrograms of the fully-glazed window for MRT and aircraft noise are shown in Fig. 3(g,h) for comparison and clearly showing the reduced passive attenuation below 300 Hz.

## Discussion

This work investigates the global attenuation potential of an ANC system for domestic open windows in attenuating common urban transportation noise. Active control of urban transportation noise is achieved while maintaining natural ventilation. Although the measured passive attenuation provided by fully closing the window always exceeded the active attenuation, the difference is only between 2.22 dB to 6.39 dB energy-average SPL. Even though the test environment was considerably ideal, the noise sources presented were measured under real-world conditions in residential buildings. It is also worth reiterating that the active system allows for natural ventilation, whereas airflow is totally restricted when the windows are closed. Unlike previously reported studies with limited aperture sizes and bulky configurations, this work has demonstrated global reduction of up to 10 dB for typical urban transportation noise for a full-sized, fully opened sliding window. The reported attenuation performance and system configuration of prior work is summarized in Supplementary Table S1.

One notable drawback of the proposed ANC system, where active control units were distributed across the aperture, is the absence of active control below 300 Hz and the restriction in control between 300 Hz to 500 Hz. This undesired tradeoff is due to the implementation of small loudspeakers to reduce the visual obstruction and to minimise the disruption to airflow. As a result of such practical design constraints, distributed-layout ANC systems for domestic façade openings would be constrained in the low-frequency range, limiting the attenuation potential of ANC in dealing with noise with dominant low-frequency content, such as jet aircraft fly-by noise or transformer noise. However, urban transportation noise with significant energy above 500 Hz, such as traffic and train noises, would be effectively mitigated by about 10 dB. Based on meta-analyses, a 10 dB reduction in equivalent transportation noise exposure level could be translated to a 7% to 17% decrease in associated health risks (i.e. hypertension, ischaemic heart diseases, including myocardial infarction)^20^.

For effective broadband noise control, the feedforward filtered-*x* least mean squares (FxLMS) approach^11,21^ is usually the algorithm of choice in real-time ANC systems^22^. More robust algorithms often incur greater computational cost, which increases exponentially with the number of channels (reference and error microphones and control sources). Since this paper prioritised practical implementation over maximum attenuation performance, the adaptive FxLMS algorithm is only utilised to generate a fixed-coefficient finite impulse response (FIR) filter in a training stage. During control, the control sources are driven by the fixed filter and the error microphones are omitted. It is worth reiterating that active control was achieved with a single reference microphone, pre-trained fixed filters and without error microphones. However, control filter coefficients derived from one bandwidth of WGN was non-optimal as demonstrated in the aircraft fly-by noise scenario. As it appears, selecting from a database of fixed-filters optimised for specific noise features – similar to noise cancelling headphones – seems to be the most practical way forward^23–26^. The methods to pre-train, select, and even switch between these fixed filters should thus be developed with low computational complexity to adhere to the causality constraints^21^, which is an ongoing area of research.

## Methods

**Figure 4:**
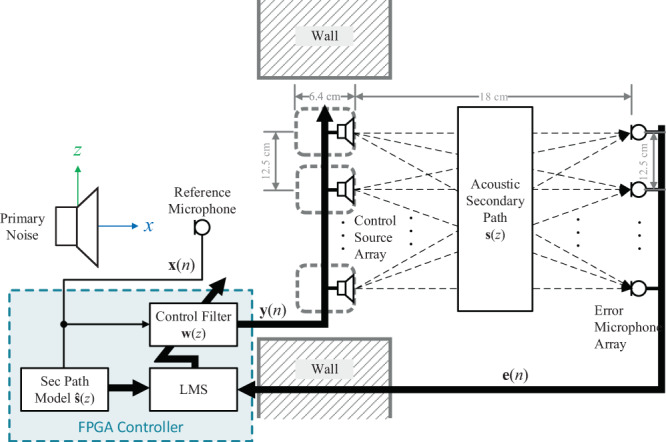
Block diagram of the active control system in the *xz*-plane, where the noise from the primary noise is sampled by the reference microphone then attenuated by the array of 24 control sources. An array of 24 error microphones is used to update the adaptive filter. All digital operations are executed in the FPGA controller.

The block diagram of the active control system is superimposed onto a *xz*-plane cross-sectional view of the window aperture, as shown in Fig. 4. To balance between computational complexity and control performance, the traditional FxLMS algorithm was employed, where the sum-of-the-squared pressures were minimised at the error microphone positions. The impinging noise from the primary source was sampled by the *J* = 1 reference microphone as the signal *x*(*n*), which is filtered by FIR filters ${\bf{w}}(z)$ to yield a set of *K* = 24 control signals **y**(*n*). The array of control loudspeakers is driven by **y**(*n*) to minimise the *M* = 24 error signals **e**(*n*) at the error microphones. Feedback from **e**(*n*) and a time-aligned *x*(*n*) updates the control filter sample-by-sample in the following update equation

1 $${\bf{w}}(n+1)={\bf{w}}(n)+\mu {\boldsymbol{x}}{\boldsymbol{{\prime} }}(n){\bf{e}}(n),$$

where ${\boldsymbol{x}}{\boldsymbol{{\prime} }}(n)={\hat{{\bf{s}}}}^{{\rm{T}}}(n)\otimes x(n)$ is the time-aligned reference signal matrix, $\hat{{\bf{s}}}(n)$ is an estimation of the actual secondary path ***s***(*z*) between the control source and the error microphone, *μ* is the step-size, and $\otimes$ denotes the Kronecker product convolution. The estimated secondary paths were derived through measurements using the least-mean-squares approach^21^.

In the training stage, the primary noise source is driven by a bandlimited WGN signal (100 Hz to 1 kHz) and the control system was allowed to adapt according to Eq. (1). Once the control filter converges to its steady state – when the noise reduction was at its maximum – the filter coefficients are stored. In the control stage, the adaptation is ceased, and the control sources are driven by the fixed coefficients as

2 $${\bf{y}}(n)=x(n){{\bf{w}}}_{BLWN}(n),$$

where ${{\bf{w}}}_{BLWN}(n)$ is the stacked coefficient vector of *K* control filters derived from the training stage with the bandlimited WGN signal. Hence, there is no feedback from the error microphones. The FxLMS algorithm was implemented efficiently on a field programmable gate array (FPGA) using the multiple-parallel-branch with folding architecture^27^. For sufficient resolution, both the control filters and secondary path estimates were set to 200 taps, which restricted the maximum sampling rate to 25 kHz on the FPGA used (﻿Xilinx Kintex-7 7K325T). To minimise the latency, 16-bit successive approximation register (SAR) analogue-to-digital (ADC) converters and 16-bit string digital-to-analogue (DAC) converters were used. Moreover, oversampling also removed the need for anti-aliasing filters, which would have added to the overall latency. The 24 control sources were also driven by low-latency class AB power amplifiers.

**Figure 5:**
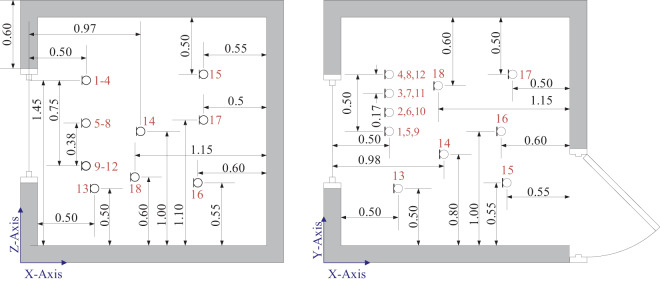
Observation microphone positions in the *xz*- and *xy*-plane. Microphones 1, and 13 to 18 are utilised in the energy-average sound pressure level calculations, and microphones 1 to 12 are used in the planar-average sound pressure level calculations^28^.

To evaluate the active control performance in the interior of the mock-up room, a total of 18 pressure microphones (GRAS 40PH, G.R.A.S., Denmark) were distributed in the interior, as depicted in Fig. 5. The global attenuation in the room interior was mainly evaluated by the energy-average SPL, *SPL*_*EA*_, from 7 microphones, as depicted in Fig. 5, given by

3 $$SP{L}_{EA}=10{\log }_{10}\left(\frac{1}{7}\mathop{\sum }\limits_{i=1}^{{N}_{EA}}{10}^{SP{L}_{TA,i}/10}\right),$$

where $SP{L}_{TA,i}$ is the time-averaged SPL of the *i*th microphone, and *i* is an element of ${N}_{EA}=\{1,13,14,15,16,17,18\}$. Microphones 1 to 12 form a rectangular plane of microphones aligned with the open aperture of the sliding window in close proximity to the plane array of 24 error microphones. These 12 microphones contribute to the planar-average sound pressure level calculations, $SP{L}_{PA}$, given by

S1 $$SP{L}_{PA}=10{\log }_{10}\left(\frac{1}{12}\mathop{\sum }\limits_{i=1}^{{N}_{PA}}{10}^{SP{L}_{TA,i}/10}\right),$$

where $SP{L}_{TA,i}$ is the time-averaged SPL of the *i*th microphone, and *i* is an element of ${N}_{PA}=\{1,2,\ldots ,12\}$.

## Conclusions

A method has been presented for attenuating urban transportation noise propagating through a full-sized fully open window in a room. A reduction of up to 10 dB has been achieved in the space and time averaged sound pressure level for typical urban transportation noise, indicating a global reduction of noise, while preserving natural ventilation. The implementation of active control in the window system was similar to a multichannel version of that used in consumer ANC headphones, with noise cancellation based on a set of pre-determined filters, tuned to eliminate noise in a specific bandwidth. Although the study indicates potential in this application, there are still issues to be addressed in future implementation studies. For instance, the strategies to select, pre-train, and switch between these fixed filters without incurring heavy computational overhead, are still being pursued. Also, without error sensors, the control performance cannot be monitored or corrected for any misadjustment, although virtual or remote microphone techniques could be explored.

## Supplementary information

Supplementary Information.

## Data Availability

The datasets generated and/or analyzed during the current study are available from the corresponding author on reasonable request.
