# Supplementary material for: Active control of broadband sound through the open aperture of a full-sized domestic window
Source: Sci Rep. 2020 Jul 9;10:10021. doi: 10.1038/s41598-020-66563-z (PMC7347542; doi:10.1038/s41598-020-66563-z)
Supplement: Supplementary file 1 — Supplementary Information. [file 41598_2020_66563_MOESM1_ESM.docx]

# Supplementary Material for “Active control of broadband sound through the open aperture of a full-sized domestic window”

**Bhan Lam**^1,*^**, Dongyuan Shi**^1^**, Woon-Seng Gan**^1^**, Stephen J. Elliott**^2^ **& Masaharu Nishimura**^3^

**^1^**School of Electrical and Electronic Engineering, Nanyang Technological University, Singapore, 639798, Singapore

^2^Institute of Sound and Vibration Research, University of Southampton, Southampton, SO17 1BJ, United Kingdom

^3^N. Lab, 1568-10, Fujie, Akashi, 673-0044, Japan

*blam002@e.ntu.edu.sg

**Simulations.** A two dimension (2D) finite element method (FEM) simulation was conducted with similar dimensions to the actual experiment setup. An *xy*-plane cross section (top view) of the window aperture is illustrated in **Supplementary Figure S1(a)**, where the aperture is partially occluded with a glass panel to emulate a simplified sliding window. The primary noise is a normally-incident plane wave that propagates in the *x*-direction. The sound propagating through the window is evaluated at 1100 points on a far-field evaluation arc with a radius of 5 m. For sufficient accuracy, the minimum element size of the FEM simulation is set to one-twelfth the wavelength of 2000 Hz. The simulation model measures 12 m by 12 m, inclusive of an encapsulating 0.5 m thick perfectly-matched layer that critically dampens the sound field to emulate a free-field condition. The glass panel is of 6 mm thickness and its density and speed of sound are set to 2180 kgm^-3^ and 5585 ms^-1^, respectively. The accuracy of the passive acoustic insulation of the glass panel derived from this simplified FEM representation has been previously verified^5^.

For a 1m wide, two-pane sliding window, the maximum open aperture size would be 0.5 m. Three active control sources are arranged in the “opened” side, as illustrated in **Supplementary Figure S1(a)**. The active control sources are implemented as line sources, which emit outward cylindrical waves instead of spherical waves. The 2D FEM model has also been deemed sufficiently accurate in representing the 3D scenario based on comparison between the propagating modes in 2D and 3D in an analogous analytical solution^29,30^. The control sources are distributed based on guidelines derived from a previous analytical and numerical study, whereby global control is achievable up to 2700 Hz when the sources are 0.125 m apart in an infinite plane array in 3D^29^ or symmetrically arranged in an unobstructed aperture in 2D^15^. Hence, this simulation determines the physical limits of active control of an asymmetrical control source placement in conjunction with passive insulation from partial glazing.

|  |  |
| --- | --- |
| (a) | (b) |
| **Supplementary Figure S1.** (**a**) Two-dimensional finite element method simulation model in the *xy*-plane to evaluate the transmission loss due to a 6 mm glass panel and a three “line source” active control system for different glazing conditions for an aperture of 1 m. (**b**) The corresponding transmission loss of the active control system with 75% (*L_o_* = 0.25 m) () and 50% (*L_o_* = 0.5 m) () glazing, and the full-glazing insulation without active control ()^28^.  | |

The acoustic insulation is formulated as the mimimisation of sound power propagating through the aperture. The sound power in the FEM model is approximated by the sum-of-the-squared pressures on the encompassing surface in the far-field^31^, i.e. evaluation arc in the FEM model. Hence, the active control sources are formulated to minimise the sum-of-the-squared pressures at the 1100 evaluation points on the arc given by the cost function in its quadratic form as

| $J=\boldsymbol{e}^{H}\boldsymbol{e}=\mathbf{q}_{s}^{H}\mathbf{A}\mathbf{q}_{s}+\mathbf{q}_{s}^{H}\mathbf{b}+\mathbf{b}^{H}\mathbf{q}_{s}+\mathbf{d}^{H}\mathbf{d}$, | (S2) |
| --- | --- |

where $\mathbf{e=d+Gu}$ is the vector of complex pressures on the evaluation arc after active control, $\mathbf{A}=\mathbf{G}^{H}\mathbf{G}$, $\mathbf{b}=\mathbf{G}^{H}\mathbf{d}$, $\mathbf{q}_{s}$ is the vector of three control source strengths in volume velocity per unit length, $\mathbf{G}$ is the matrix of transfer functions between all control sources and each evaluation point, $\mathbf{d}$ is the vector of complex pressures due to the primary source at the evaluation points, and ${}^{H}$ is the Hermitian transpose operator. Hence, the optimal source strengths were calculated by equating the derivative of (S1) to zero to give

| $\mathbf{q}_{s}=-\left( \mathbf{G}^{H}\mathbf{G}+\beta\mathbf{I} \right)^{-1}\mathbf{G}^{H}\mathbf{d,}$ | (S3) |
| --- | --- |

where $\beta$ is the regularisation parameter that was set to a small value to alleviate the ill-conditioning of the matrix, and $\mathbf{I}$ is an identity matrix. Both the passive and active attenuation is evaluated by the transmission loss through the aperture evaluated at the evaluation arc as

| $Transmisson Loss=-10\log_{10} \frac{\mathbf{e}^{H}\mathbf{e}}{\mathbf{d}^{H}\mathbf{d}}.$ | (S4) |
| --- | --- |

The passive transmission loss of full glazing with a 6 mm thick glass panel is illustrated by the dashed purple line in **Supplementary Figure S1(b)**. At 50% glazing, the largest aperture opening for a two-pane sliding configuration, active control performance exceeds that of full glazing insulation up to 300 Hz and rolls off with increasing frequency to plateau at around 1000 Hz to 10 dB. In contrast, a minimum of 75% glazing is required for active control performance to exceed that of full glazing insulation up about 1000 Hz, as shown by the dark green solid line in **Supplementary Figure S1(b)**. It is also worth noting the contribution of passive insulation from the partially open window panel is minimal, as illustrated by the light blue and green solid lines for 50% and 75% glazing respectively. Therefore, theoretical transmission loss of more than 10 dB is expected up to 1000 Hz with three active control “line” sources at 50% glazing.

**Summary of prior work.** In general, active control sources are either arranged around the boundary or distributed uniformly across the entire aperture. The reported active attenuation performance of prior work for open apertures are summarised in **Supplementary Table S1**. From the table, it can be observed that boundary layout strategies function well with limited opening sizes. It has also been established that the attenuation performance of the boundary layout is dependent on the size of the aperture rather than the number of control sources^32^. This limitation is evident in the observations by Brüggemann et al., whereby attenuation deteriorated for a window which was 0.5 times longer even after a 2.5 times increase in the number of control sources. Although the distributed layout has higher scalability to larger apertures, the largest opening size in a window-like configuration has been limited to just 25 × 25 cm^2^ by Murao and Nishimura in 2012. The work presented here demonstrates the scalability of the distributed layout strategy for a full-sized, fully-opened sliding window. Extensive work by Wang et al. on the control of noise propagating through an aperture of a baffled rectangular cavity is included for comparison. However, the physical nature of the baffled rectangular cavity is fundamentally different. The active control system is optimized to minimize the noise propagating out of the opening.

| **Supplementary Table S1**: Summary of reported attenuation performance and system configurations for active noise control systems designed for windows and open apertures^5,28^. | | | | | | | |
| --- | --- | --- | --- | --- | --- | --- | --- |
| Author | Layout | Type | Window Dimensions (W×H cm) | Opening Size | No. of Control Sources | Type of Noise | Reduction  (Global/ Local) |
| Window | | | | | | | |
| Ise 2005^33^ | Distributed | Open Aperture | Not  Stated | Not Stated | 16 | BLWN  (0.2 to 0.7 kHz) | 10 dB (Local) |
| Murao 2012^8^ | Distributed | Open Aperture | 25 × 25 | 25 × 25 | 4 | BLWN  (0.5 to 2kHz) | 10-15 dB (Global) |
| Kwon 2013^7^ | Boundary | Open Aperture | 30 × 30 | 30 × 30 | 8 | BLWN  (0.4 to 1 kHz) | Up to 10 dB (Global) |
| Paimes 2014^34^ | Boundary | Tilt Window (Hopper) | 56 × 142 | 5cm Gap 2° Tilt | Not stated | Real aircraft pass-by (0.2 to 0.16 kHz) | 3 dB  (Global) |
| Carme 2016^35^ | Boundary | Sliding Window | 75 × 75 | 13 × 75 | 5 | Traffic Noise (<300 Hz) | 15.5 dB (Not Stated) |
| Hanselka 2016^36^  Eder 2017^37^ | Boundary | Tilt Window (Hopper) | 91 × 91 | Not stated | 8 | BLWN  (0.1 to 1 kHz) | 13 dB  (Local) |
| Brüggemann^38^ | Boundary | Tilt Window (Hopper) | 95 × 132.5 | Not stated | i. 14  ii. 20 | BLWN  (0.1 to 1 kHz) | i. 5.5 dB (Local)  ii. 10.0 dB (Local) |
| This work | Distributed | Sliding Window | 100 × 100 | 45 × 93 | 24 | i. BLWN*  ii. Traffic*  iii. Train*  iv. Aircraft*  *(0.1 to 1 kHz) | i. 8.8 dB^  ii. 8.67 dB^  iii. 10.14 dB^  iv. 7.51 dB^  ^(global) |
| The opening of the baffled rectangular cavity | | | | | | | |
| Wang 2015, 2016^17,39^ | Distributed | Open Aperture | - | 43 x 67 | 6 | BLWN  (<0.5 kHz) | ~15 dB (Global) |
| Wang 2017^6^ | Boundary | Open Aperture | - | 43 x 67 | 8 | BLWN  (<1 kHz) | 10 dB  (Local, 0.2 m around error points) |
| Wang 2017^6^ | Boundary | Open Aperture | - | 43 x 67 | 32 | Tonal  (<1 kHz) | ~20dB (Global) |
| Wang 2017^6^ | Distributed | Open Aperture | - | 43 x 67 | 32 | Tonal  (<1 kHz) | ~20dB (Global) |

## References

29. Elliott, S. J., Cheer, J., Lam, B., Shi, C. & Gan, W. A wavenumber approach to analysing the active control of plane waves with arrays of secondary sources. *J. Sound Vib.* **419**, 405–419 (2018).

30. Elliott, S., Cheer, J., Lam, B., Shi, C. & Gan, W. Controlling Incident Sound Fields With Source Arrays in Free Space and Through Apertures. in *Proceedings of the 24th International Congress on Sound and Vibration* (ed. Gibbs, B. M.) 1–7 (2017).

31. Nelson, P. A. & Elliott, S. J. *Active control of sound*. (Academic Press, 1992).

32. Wang, S., Tao, J., Qiu, X. & Pan, J. A boundary secondary source arrangement for a virtual sound barrier system at a cavity opening. in *INTER-NOISE and NOISE-CON Congress and Conference Proceedings* 1733–1740 (2017).

33. Ise, S. The Boundary Surface Control Principle and Its Applications. *IEICE Trans. Fundam. Electron. Commun. Comput. Sci.* **E88**-**A**, 1656–1664 (2005).

34. Pàmies, T., Romeu, J., Genescà, M. & Arcos, R. Active control of aircraft fly-over sound transmission through an open window. *Appl. Acoust.* **84**, 116–121 (2014).

35. Carme, C., Schevin, O., Romerowski, C. & Clavard, J. Active Noise Control Applied to Open Windows. in *INTER-NOISE and NOISE-CON Congress and Conference Proceedings* 3058–3064 (2016).

36. Hanselka, J., Jukkert, S. & Sachau, D. Experimental Study on the Influence of the Sensor and Actuator Arrangement on the Performance of an Active Noise Blocker for a Tilted Window. in *INTER-NOISE and NOISE-CON Congress and Conference Proceedings* 3046–3057 (2016).

37. Eder, J., Hanselka, J. & Sachau, D. Experimental study on the effect of the number of system components of an active noise blocker on the global noise reduction. in *INTER-NOISE and NOISE-CON Congress and Conference Proceedings* 5298–5309 (2017).

38. Brüggemann, H., Hanselka, J. & Sachau, D. Experimental study on the effects of increasing number of system components and sampling rate of an active noise blocker for a tilted window. in *INTER-NOISE and NOISE-CON Congress and Conference Proceedings, InterNoise19* 3829–3838 (Institute of Noise Control Engineering, 2019).

39. Wang, S., Tao, J. & Qiu, X. Performance of a planar virtual sound barrier at the baffled opening of a rectangular cavity. *J. Acoust. Soc. Am.* **138**, 2836–2847 (2015).
